# Fundamental Perceptual Characterization of an Integrated Tactile Display with Electrovibration and Electrical Stimuli

**DOI:** 10.3390/mi10050301

**Published:** 2019-05-03

**Authors:** Seiya Komurasaki, Hiroyuki Kajimoto, Hiroki Ishizuka

**Affiliations:** 1 Division of Intelligent Mechanical Systems Engineering, Graduate School of Engineering, Kagawa University, 2217-20 Hayashi-cho, Takamatsu, Kagawa 761-0396, Japan; s15t421@stu.kagawa-u.ac.jp; 2 Department of Informatics, University of Electro-Communications, 1-5-1 Chofugaoka, Chofu, Tokyo 182-8585, Japan; kajimoto@kaji-lab.jp; 3 Division of Bioengineering, Department of Mechanical Science and Bioengineering, Graduate School of Engineering Science, Osaka University, 1-3 Machikaneyama, Toyonaka, Osaka 560-8531, Japan

**Keywords:** tactile display, electrode array, electrical stimulus, electrovibration stimulus, multiple tactile stimuli

## Abstract

Tactile displays have been widely studied for many decades. Although multiple tactile stimuli are more effective to improve the quality of the presented tactile sensation, most tactile displays provide a single tactile stimulus. An integrated tactile display with electrovibration and electrical stimuli is proposed herein. It is expected that vibrational friction, pressure and vibration can be presented at the same time through the tactile display. Also, these stimuli only require electrodes for stimulation. Therefore, the tactile display can be easily miniaturized and densely arrayed on a substrate. In this study, a tactile display is designed and fabricated using the micro-fabrication process. Furthermore, the display is evaluated. First, the relationship between a single stimulus and the perception is investigated. The electrovibration and electrical stimuli have a frequency dependence on perception. Second, whether the multiple stimuli with the electrovibration and electrical stimuli are perceivable by the subjects is also evaluated. The results indicate that the multiple tactile stimuli are perceivable by the subjects. Also, the possibility that the electrovibration and electrical stimuli affect each other is confirmed.

## 1. Introduction

Tactile displays provide a tactile stimulus to users and have been widely studied for several decades to improve the quality of a presented tactile sensation and their applications. Tactile displays stimulate tactile mechanoreceptors, which perceive a tactile stimulus through the skin and provide tactile sensation to users. Tactile displays can provide not only a vibrational stimulus for simple tactile feedback [1,2] but also realistic tactile sensation for a virtual surface with detailed signal control [3,4]. According to the stimulus principles, tactile displays can be categorized into mechanical tactile displays and electrical tactile displays.

Most mechanical tactile displays provide a tactile stimulus such as displacement or vibration with actuators. For example, Lévesque et al. developed a tactile display with an array of piezo actuators [5]. They experimentally confirmed that the developed tactile display was able to provide virtual braille dot patterns with the lateral displacement of actuators. Zhang and Follmer combined a linear actuator and an electrostatic actuator [6]. They densely arrayed them to develop a high spatial resolution tactile shape display. Kosemura et al. developed a micro-fabricated tactile display with an array of piezo actuators [7]. They experimentally investigated the relationship between the vibration condition of actuators and the presented virtual surface through the tactile display. Zhao et al. developed a compact tactile display with an array of shape-memory alloy wires to present braille dot patterns [8]. Singh et al. developed a portable tactile display with encapsulated magnetic fluid, which has the characteristic of being attracted toward the magnetic field [9]. Hoshi et al. applied ultrasonic radiation to tactile feedback [10]. The developed tactile display consisted of an array of ultrasound transducers and was able to provide acoustic radiation pressure without any contact. In recent years, Hasegawa and Shinoda proposed a method to provide vibrational sensation to users with ultrasonic radiation [11]. Strong and Troxel developed an electrovibration tactile display based on frictional force modulation with an electrostatic force between a sliding finger pad and an electrode [12]. The electrovibration tactile display can provide vibrational friction induced by the modulated frictional force. The electrovibration tactile display only requires an electrode and an insulator layer. Therefore, the devices are thin and not bulky, as compared with the devices based on other principles. Bau et al. integrated an electrovibration tactile display into a touchscreen to provide tactile feedback through the touchscreen [13]. They also proposed a method to add the electrovibration feedback to any object [14].

Electrical tactile displays stimulate tactile mechanoreceptors inside the skin using current stimulation. These displays can provide tactile sensation such as vibration and pressure [15]. In comparison with mechanical tactile displays, electrical tactile displays have a simple structure because they require only electrodes for stimulation. Kaczmarek and Haase developed an electrical tactile display and investigated the relationship between voltage waveforms and the perception of stimulus patterns [16]. Kajimoto developed an electrical tactile display consisting of an array of almost 1500 electrodes to stimulate the whole palm [17]. Kitamura et al. developed a needle-type electrical tactile display for low-voltage stimulation [18].

The mentioned mechanical and electrical tactile displays can provide only a single tactile stimulus. It is known that human beings perceive tactile sensation by integrating signals from tactile mechanoreceptors [19]. A combination of tactile stimuli is effective to improve the quality of the perceived tactile sensation, because many mechanoreceptors inside the skin can be stimulated at the same time with multiple tactile stimuli. For this purpose, the realization of a tactile display that can provide multiple tactile stimuli is required to improve the quality of the presented tactile sensation through the tactile display. However, only a few groups have dealt with tactile displays for multiple tactile stimuli. Yem and Kajimoto integrated an electrical tactile display and a vibrator to develop a wearable tactile display [3]. Pyo et al. developed an integrated tactile display with an electrode for the electrovibration stimulus and an electrostatic vibrator [20]. Ryu et al. integrated an electrovibration tactile display and vibrators [21]. They revealed the relationship between the multiple tactile stimuli and perception. A tactile display with magnetic actuators and a Peltier element was developed in [22]. The tactile display was able to provide both vibration and thermal stimulus. We previously developed an integrated tactile display with an electrovibration tactile display and a Peltier element as the application of a flexible tactile display [23]. The problem with the mentioned tactile displays is their bulkiness, due to the use of mechanical actuators in such displays. The bulkiness of a tactile display makes it difficult to be integrated into other devices and restricts its application area.

**Figure 1:**
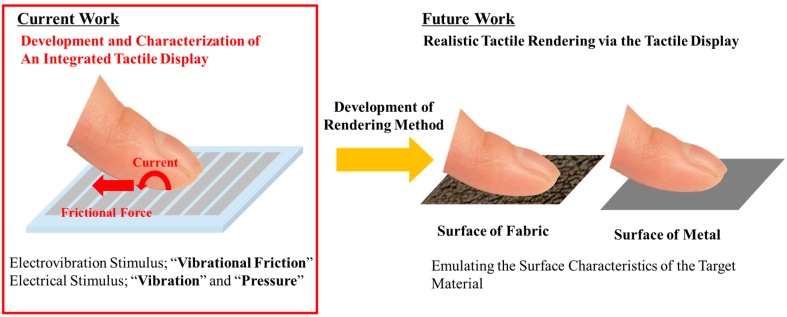
Concept of the proposed tactile display. The tactile display can provide both electrovibration stimulus and electrical stimulus with an array of electrodes. In this study, we revealed that the multiple stimuli were able to be perceived by the subjects.

In this study, we propose a novel integrated tactile display based on the principles of the electrovibration and electrical stimuli, as shown in Figure 1. The electrovibration and electrical stimuli have different characteristics. Therefore, vibrational friction, pressure and vibration can be provided to users at the same time by combining these principles. Additionally, as stated earlier, both electrovibration and electrical stimuli only require small electrodes for stimulation. The integrated tactile display can be easily miniaturized, made thin and has the advantage of easy implementation. Therefore, it can be expected that the integrated tactile display is able to be integrated into other information devices and widens the application of the tactile feedback. Also, the stimuli resolution of the integrated tactile display can be easily increased by arraying the electrodes densely. This also contributes to the improvement in the quality of the presented tactile sensation. We previously developed an integrated tactile display with electrovibration and electrical stimuli [24]. In that tactile display, the electrovibration stimulus was applied through a sheet-type slider as frictional force and the application was limited to wearable uses. The tactile display proposed in this study can directly provide electrovibration and electrical stimuli to the finger pad through an array of flat electrodes and is preferred for applications such as touchscreens. In this study, we designed and fabricated the integrated tactile display using the micro-fabrication process. We conducted a sensory experiment to characterize each stimulus with subjects. Finally, we also evaluated whether the multiple tactile stimuli were able to be perceived by subjects because the combination of these tactile stimuli has never been investigated.

## 2. Principle and Design

**Figure 2:**
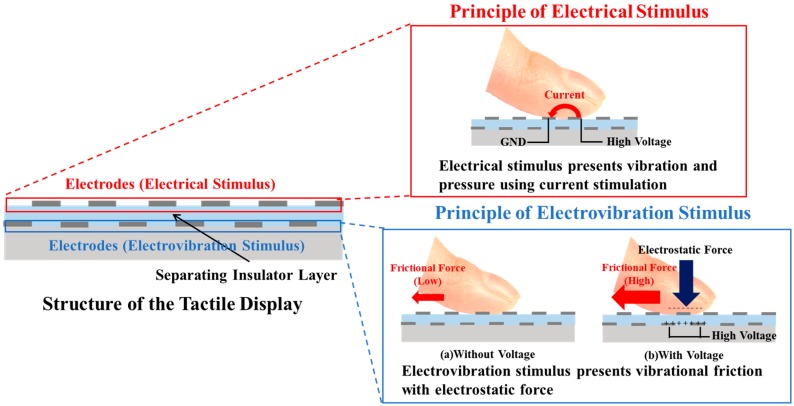
Principle and structure of the proposed tactile display. Electrodes for electrical stimulus are located on the surface of the tactile display. The electrodes for the electrovibration stimulus were formed under the electrodes for the electrical stimulus.

Figure 2 shows the working principle and the structure of the integrated tactile display. Electrodes for the electrovibration and electrical stimuli were formed on a substrate, as shown in Figure 2 (left side).

On the surface of the tactile display, electrodes for the electrical stimulus were provided. Normally, two electrodes are used for electrical stimulation. One electrode is connected to a high voltage and other electrode is grounded. When a user touches the electrodes, the electrodes are electrically connected because of the electrical conductivity of the contacting skin (Figure 2, upper right). Then, current flows through the skin and stimulates tactile mechanoreceptors such as Merkel’s disks and Meissner’s capsules, which are located near the surface of the skin, and the user can perceive tactile sensation such as vibration and pressure. The intensity and frequency of the electrical stimulus can be controlled by controlling the applied current waveform. The electrodes for the electrovibration stimulus are formed under the electrodes for the electrical stimulus. The principle of the electrovibration is shown in Figure 2 (bottom right). Electrovibration requires an insulator and electrodes for the stimulation and is based on an electrostatic force generated by the polarization of skin. The polarized skin and electrodes attract each other, with Coulomb’s law and an electrostatic force applied to the skin. The electrostatic force is not strong enough for users to perceive the attractive force. However, the electrostatic force is strong enough to modulate the resulting frictional force to a sliding finger pad. Without voltage, when a user slides a finger pad on the surface of the tactile display, no external force is applied to the finger pad. Then, the user perceives a smooth surface. On the other hand, with voltage applied to electrodes, the electrodes are charged positively and the sliding finger pad is charged negatively because of the dielectric polarization of the separating insulator layer. As a result, an electrostatic force is applied to the sliding finger pad. The finger pad is attracted toward the electrodes and the resulting frictional force to the finger pad is increased. The electrostatic force and resulting frictional force are expressed as follows [25]:

(1) $$F=\frac{A\varepsilon \varepsilon_{0}}{2}{\left(\frac{V^{\prime }(t)}{d}\right)}^{2}$$

(2) $$F^{\prime }=\mu \left(F+N\right)=\mu \left(\frac{A\varepsilon \varepsilon_{0}}{2}{\left(\frac{V^{\prime }\left(t\right)}{d}\right)}^{2}+N\right)$$

where *F* is the electrostatic force of the finger pad, *ε* is the relative permeability of the stratum corneum, *ε*_0_ is the vacuum permeability, *A* is the overlap area between the finger pad and the electrode, *V*′(*t*) is the applied voltage across the stratum corneum, *d* is the thickness of the stratum corneum, *F*′ is the resulting frictional force, *µ* is the frictional coefficient and *N* is the normal force toward the surface. The equations consider the effects of the skin condition, the contacting condition and the device on the electrostatic force and resulting frictional force, because the voltage across the stratum corneum represents the resulting voltage modulated by the mentioned effects. We consider that the equations well represent the actual condition. Normally, a periodic voltage is applied to the electrovibration tactile display. This results in a periodic change in the resulting frictional force. The entire finger pad is periodically vibrated and tactile mechanoreceptors such as Pacinian corpuscles, which are located deep within the finger pad, are stimulated. Thus, the user can perceive vibrational friction through the electrovibration tactile display. Equations (1) and (2) indicate that the intensity and frequency of the electrovibration stimulus can be controlled by the applied voltage waveform. The user can perceive both electrical and electrovibration stimuli, by sliding the finger pad on the proposed tactile display. By controlling the current to the electrodes for the electrical stimulus and the voltage to the electrodes for the electrovibration stimulus, the intensity and frequency of the provided multiple stimuli can be controlled.

**Figure 3:**
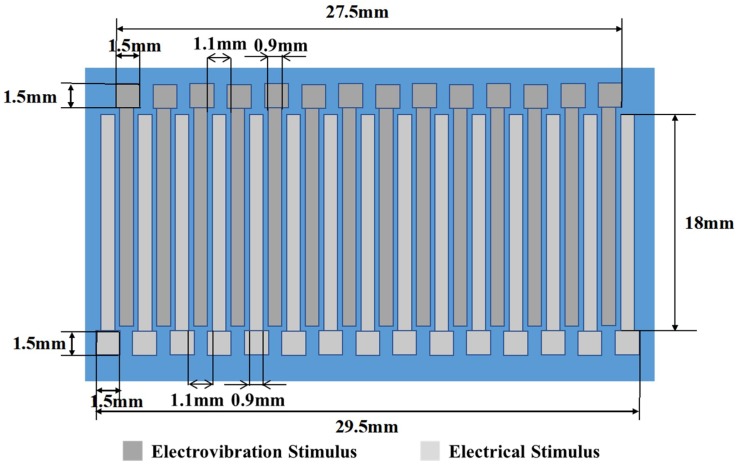
Design of the tactile display. The width and length of electrodes for each stimulus are 0.9 mm and 18 mm, respectively. The separation distance between the electrodes for each stimulus is 1.1 mm. Fifteen electrodes for each stimulus are arranged on a substrate.

Figure 3 shows the design of the proposed tactile display. The width and length of electrodes for each stimulus were 0.9 mm and 18 mm, respectively. The electrode designs were determined on the basis of the results of related studies, considering the spatial stimulation [26,27]. A pad for electrical connection was formed at the end of the electrode. The area of the pad was 1.5 mm × 1.5 mm. The separation distance between electrodes for each stimulus was 1.1 mm. The electrodes for the electrovibration stimulus and the electrodes for electrical stimulation were alternately arranged. The thickness of the separating insulator layer was 4 μm. A total of 15 electrodes for each stimulus were formed on a glass substrate.

## 3. Fabrication Process

**Figure 4:**
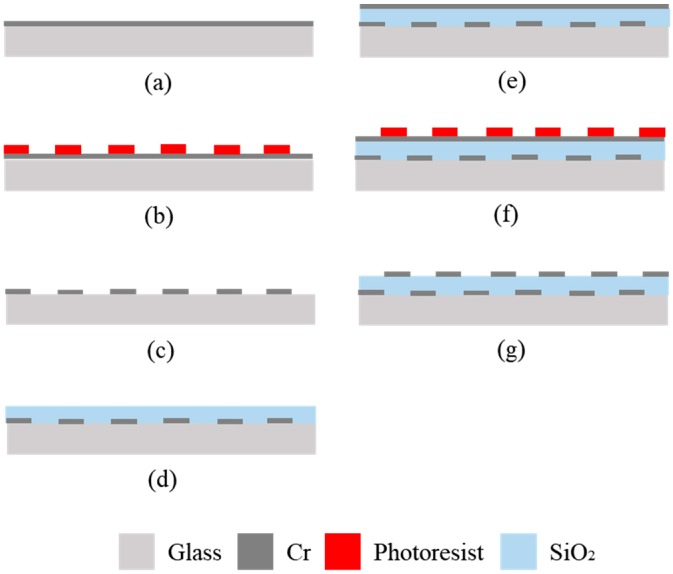
Fabrication process of the tactile display. (**a**) Cr layer deposition; (**b**) photoresist patterning; (**c**) Cr etching; (**d**) SiO_2_ deposition; (**e**) Cr layer deposition; (**f**) photoresist patterning; (**g**) Cr etching.

**Figure 5:**
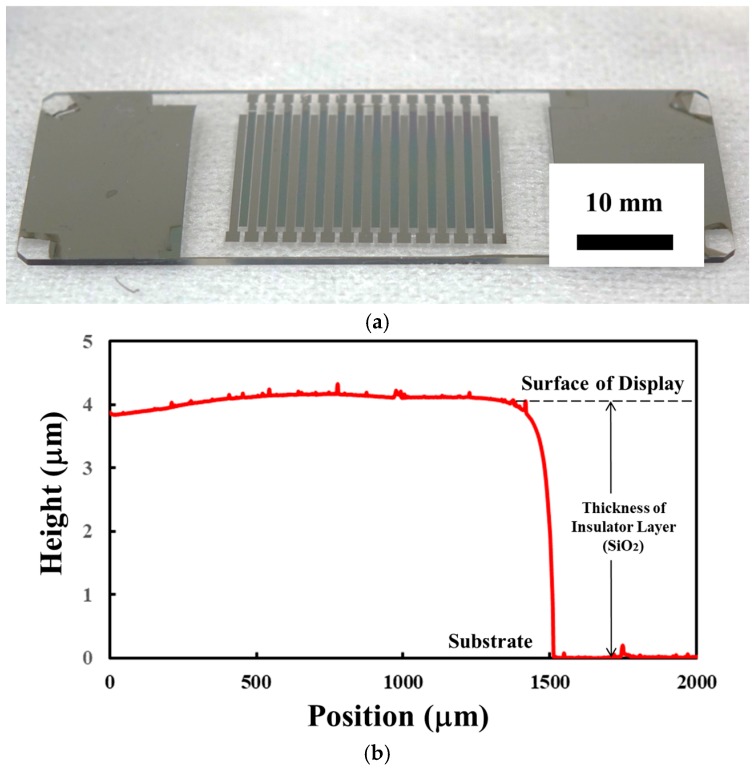
Fabrication results. (**a**) Photograph of the fabricated tactile display. (**b**) The measured thickness of the insulator layer.

Figure 4 shows the fabrication process of the integrated tactile display. A glass plate was dipped into hydrolysis with sulfuric acid for 5 min to remove any remaining organic matter. O_2_ plasma treatment was given for 10 min to strongly adhere Cr to the glass plate. Cr was deposited on the glass plate for 7 min, as shown Figure 4a. The resulting thickness of the Cr layer was 100 nm. A positive photoresist layer was formed on the Cr layer with spin coating at 3000 rpm. The photoresist was exposed to UV light with a photo mask. The exposed photoresist was selectively dissolved with a photoresist developer, as shown in Figure 4b. The bare part of the Cr layer was dissolved with a Cr etching solution to form an electrode pattern for the electrovibration stimulus. The remaining photoresist was dissolved through hydrolysis with sulfuric acid, as shown in Figure 4c. SiO_2_ was deposited on the glass plate for 400 min to form an insulator layer, as shown in Figure 4d. The resulting thickness of the insulator layer was almost 4 μm. Cr was deposited again and the resulting thickness of the Cr layer was 100 nm, as shown in Figure 4e. The photoresist layer was formed on the Cr layer through spin coating. The patterned photoresist was obtained with UV light exposure and a photoresist developer, as shown in Figure 4f. Finally, the Cr layer was etched with a Cr etching solution to form an electrode pattern for the electrical stimulation, and the remaining photoresist was removed through hydrolysis with sulfuric acid, as shown in Figure 4g. Figure 5a is a photograph of the fabricated tactile display. Figure 5b shows the measured thickness of the insulator layer with a stylus profiler. The insulator layer with a thickness of almost 4 μm was formed on the glass substrate.

## 4. Experimental Procedure

To characterize the fabricated tactile display, we conducted sensory experiments with one female and eight male subjects (average age: 22.1 years, SD: 1.0). This experiment was approved by the Research Ethics Committee of Kagawa University (30-005).

### 4.1. Experimental Setup

**Figure 6:**
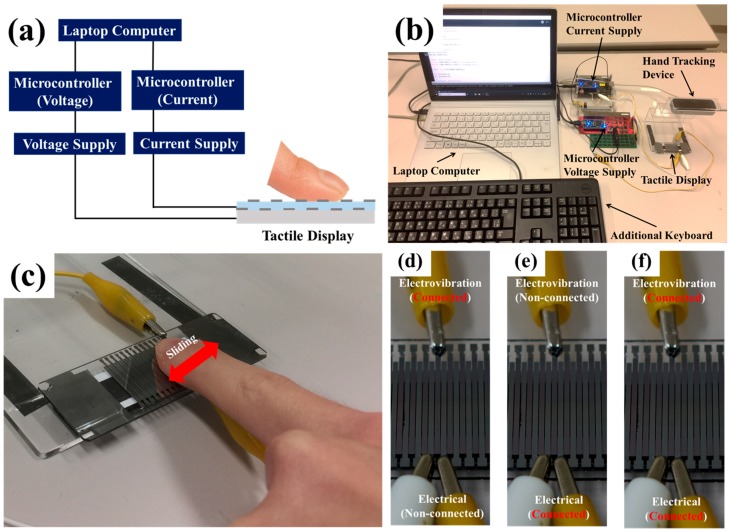
(**a**) Schematic illustration of the experimental setup. (**b**) Actual photograph of the experimental setup. (**c**) Actual photograph of the experiment. (**d**) Electrode connection for the electrical stimulus evaluation. (**e**) Electrode connection for the electrovibration stimulus evaluation. (**f**) Electrode connection for the multiple stimuli evaluation.

Figure 6a shows the schematic illustration of the experimental setup. The experimental setup consisted of a laptop computer (Surface Book Pro, Microsoft Corp., Redomond, WA, USA), microcontrollers (mbed LPC 1768, ARM Ltd., Cambridge, UK), a high current supply for the electrical stimulus (MHV 12-300S10P, Bellnix Co., Ltd., Saitama, Japan), a high voltage power supply for the electrovibration stimulus (MHV 12-1.0k2000P, Bellnix Co., Ltd., Saitama, Japan), an additional keyboard (KB212-B, Dell Inc., Round Rock, TX, USA), a hand tracking system (LEAP MOTION, LEAP MOTION Inc., San Francisco, CA, USA) and the fabricated tactile display. An actual photograph of the experimental setup is shown in Figure 6b. The values of the current for the electrical stimulus and the voltage for the electrovibration stimulus were changeable through a control system on the laptop. The values were increased/decreased by pressing assigned buttons on the keyboard. A control signal was sent to the microcontroller according to the control system and the microcontroller controlled the voltage or current waveform according to the signal. The value of the peak voltage provided by the voltage power supply for the electrovibration stimulus was varied from 0 V to 600 V. The average voltage increase/decrease was 2.945 V/pressing. Furthermore, the value of the peak current provided by the current supply for the electrical stimulus was varied from 0 mA to 5 mA. The average current increase/decrease was 0.025 mA/pressing. Before the experiments, we instructed the subjects to slide their dominant finger pad with a speed of 50 mm/s using the hand tracking device. The average room temperature and humidity were 21.8 °C and 36.5%, respectively. Figure 6c shows a photograph of the actual experiment.

### 4.2. Evaluation for a Single Tactile Stimulus

First, we evaluated whether the fabricated tactile display was able to successfully provide the electrovibration and electrical stimuli with the designed electrodes. We experimentally evaluated the minimal values of the voltage for the electrovibration stimulus and the current for the electrical stimulus to reveal the minimal stimulus conditions and the trends of the perception with the fabricated tactile display. In this experiment, the subjects slid their dominant finger pad on the tactile display from side to side and adjusted the value of the voltage for the electrovibration stimulus by pressing a button. We asked them to note the minimal value of the voltage where they were able to perceive the electrovibration stimulus. One electrode for the electrovibration stimulus was connected to the voltage power supply, as shown in Figure 6d. The voltage waveform was square and the duty cycle of the voltage was 20%. The perceivable duty cycle was selected from the related study [23]. The frequency of the voltage was selected from 5 Hz, 20 Hz, 80 Hz, 160 Hz and 320 Hz. Each frequency was evaluated twice and a total of 10 trials were conducted. After the evaluation for the electrovibration stimulus, we also conducted trials for the evaluation of the electrical stimulus. In this experiment, the subjects slid their dominant finger pad on the tactile display from side to side and adjusted the value of the current for the electrical stimulus by pressing a button. We asked them to note the minimal value of the current where they were able to perceive the electrical stimulus. In this experiment, two electrodes for the electrical stimulus were connected to the current supply, as shown in Figure 6e. The pulse width of the current was fixed at 200 µs. We determined the perceivable pulse width from the related study [28]. The frequency of the current was selected from 5 Hz, 20 Hz, 80 Hz, 160 Hz and 320 Hz. Each frequency was evaluated twice and a total of 10 trials were conducted.

### 4.3. Evaluation for Multiple Tactile Stimuli

Next, we evaluated whether the multiple tactile stimuli with electrovibration and electrical stimuli were perceivable by the subjects. In this experiment, we evaluated the minimal value of the current for the electrical stimulus with the electrovibration stimulus applied, to confirm that the electrical stimulus was successfully added to the electrovibration stimulus. Before the experiment, the subjects experienced the electrical stimulus to distinguish it from the electrovibration stimulus. In this experiment, the electrovibration stimulus was provided as a base tactile stimulus. The voltage waveform for the electrovibration stimulus was square and the duty cycle was 20%. The peak voltage where all subjects were able to perceive the electrovibration stimulus under the selected frequencies was approximately 330 V. The subjects slid their dominant finger pad on the tactile display from side to side and adjusted the value of the current for the electrical stimulation. We asked them to note the minimal value of the current where they were able to perceive the electrical stimulus. In this experiment, two electrodes for the electrical stimulus were connected to the current supply for the electrical stimulus, and one electrode for the electrovibration stimulus between the electrodes for the electrical stimulus was connected to the voltage power supply, as shown in Figure 6f. The pulse width of the current was fixed at 200 µs. The frequencies of each stimulus were 20 Hz (low), 80 Hz (middle) and 320 Hz (high). We evaluated the combination of three frequencies for the electrovibration stimulus and three frequencies for the electrical stimulus. Each combination was evaluated twice. A total of 18 trials were conducted.

## 5. Experimental Results

### 5.1. Evaluation for a Single Tactile Stimulus

**Figure 7:**
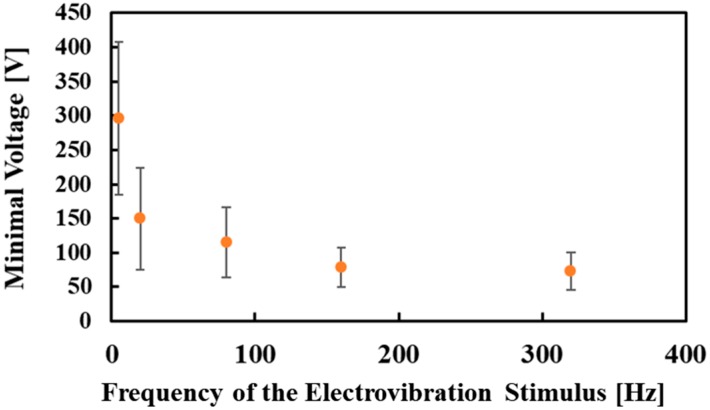
The relationship between the frequency of the electrovibration stimulus and the minimal voltage. The minimal voltage decreased with a decrease in the frequency. The trend was similar to the reported trends.

**Figure 8:**
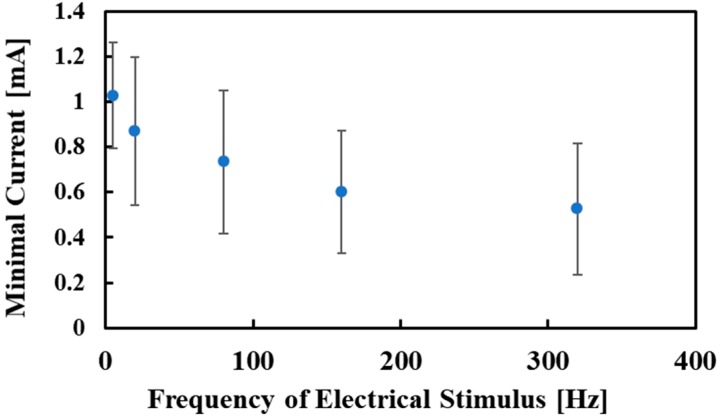
The relationship between the frequency of the electrical stimulus and the minimal current. The minimal current decreased with a decrease in the frequency. The minimal current was also affected by the frequency.

Figure 7 and Figure 8 show the experimental results for electrovibration and electrical stimuli, respectively. The average values were plotted. A subject was not able to perceive the electrovibration stimulus with a frequency of 5 Hz in a trial. Thus, the number of the obtained threshold voltages for the electrovibration stimulus with a frequency of 5 Hz was 17. The minimal values of the voltage for the electrovibration stimulus was decreased by increasing the frequency of the applied voltage. The values were not largely changed under high-frequency conditions. The obtained trend was similar to the results of related studies [25,26]. One considerable reason for this trend is the attenuation of the applied voltage induced by the skin and devices [25]. Another reason is the perception of human beings. Pacinian corpuscles, which are the target of the electrovibration stimulus, are not sensitive to low-frequency vibration [29]. Therefore, the subjects were not sensitive to the electrovibration stimulus, which was the vibrational friction, under low-frequency conditions. The minimal values were higher compared with the related studies [25,26]. In this study, the finger pads were not grounded because some subjects perceived pain in trial experiments for the electrical stimulus under the grounded state. The ungrounded state of the finger pads caused an increase in the minimal values. Also, the ungrounded state of the finger pads might increase the standard deviations. In our previous study, the standard deviations were lower than those of this study [26]. The finger pads were grounded and the voltages between the subjects and the device were almost constant in the previous study. In this study, the voltages between the subjects and the device were not controlled. This resulted in high standard deviations. We also found that the difference in the skin conditions among the subjects affected the threshold voltages and the standard deviations. The minimal values of the current for the electrical stimulus were also decreased by increasing the frequency. The trend was different from the related study, although the finger pad was fixed in the related study [18]. In the related study, the minimal values of the current were not largely changed by the frequency of the applied current. We considered that the sliding of the finger pad affected the trend of the minimal values of the current. The current was applied to the finger pad only when the pad was overlapped with electrodes. In this experiment, the sliding of the finger pad decreased the time when the finger pad and electrodes were contacted. Therefore, under low-frequency conditions, the number of the electrical stimuli applied to the finger pad decreased and the subjects were not sensitive to the electrical stimulus. The standard deviations were relatively high. The related study showed that the skin impedance conditions were different among the subjects [27]. The relatively high standard deviations in the experiment using a flat electrode tactile display were shown in the related study [18]. In this experiment, the difference in the skin conditions also affected the minimal values of the current and the standard deviations were increased. We considered that the electrical stimulus was not a stable stimulus and a method to stabilize the intensity of the electrical stimulus was required.

### 5.2. Evaluation for Multiple Tactile Stimuli

**Figure 9:**
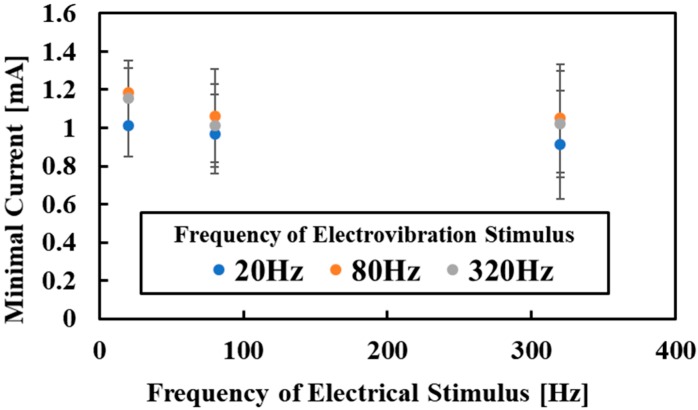
Experimental results for multiple tactile stimuli. Multiple tactile stimuli were perceived by the subjects. The frequency dependence of the perception on the electrical stimulus was smaller than that of the single electrical stimulus.

Figure 9 shows the experimental results for multiple tactile stimuli. The average values were plotted. The subjects successfully perceived the electrical stimulus even though the electrovibration stimulus was applied. The minimal values of the current were increased, compared with the values for the single electrical stimulus, as shown in Figure 8. These results indicate that the electrovibration stimulus masked the perception of the electrical stimulus and the subjects required larger currents to perceive the electrical stimulus. Also, the frequency of the electrovibration stimulus might have affected the perception of the electrical stimulus, because the minimal current tended to be relatively low under the low-frequency electrovibration stimulus. For the single electrical stimulus, we confirmed the frequency dependence of the perception. However, in this experiment, it seemed that the effect of the frequency of the current on the minimal values was smaller than that of the single electrical stimulus. Therefore, there is a possibility that the electrovibration stimulus and the electrical stimulus interact with each other. Sometimes, the subjects did not perceive the electrovibration stimulus when the intensity of the electrical stimulus was too high. The high intensity of the electrical stimulus also masked the electrovibration stimulus. The relationship between the intensities of two stimuli and the perception should be revealed in detail to utilize multiple tactile stimuli. Further investigations are required to characterize multiple tactile stimuli with the electrovibration stimulus and the electrical tactile stimulus.

## 6. Conclusions

To develop a tactile display for multiple tactile stimuli, in this study we designed and fabricated an integrated tactile display using electrovibration and electrical stimuli with the micro-fabrication process. The width and length of the electrodes for each stimulus were 0.9 mm and 18 mm, respectively. The electrodes were densely arrayed on a glass substrate. In the first experiment, we evaluated the relationship between the single stimulus and the perception with the designed electrodes. The obtained trend for the electrovibration stimulus was similar to the previously reported trends. For the electrical stimulus, the trend was different from the reported results because of the sliding of the finger pad. We also evaluated whether the multiple stimuli with the electrovibration stimulus and the electrical stimulus were able to be perceived. The subjects successfully perceived the electrical stimulus with the electrovibration applied. Also, the experimental results indicated that the electrovibration stimulus affected the perception of the electrical stimulus. In future studies, we will evaluate the relationship between the multiple tactile stimuli and the perception in detail, because the relationship between the multiple tactile stimuli and the perception is important to determine the applicable driving condition of the tactile display. In the experiments, the age range and gender of the subjects were not considered. The experimental results revealed the perception of males in their 20s to the tactile stimuli. In order to consider real-world cases, we will evaluate the effects of the age range and gender of subjects in future work, because these factors may have effects on skin characteristics. Also, we will develop methods to provide the electrovibration stimulus and electrical stimulus stably. Additionally, we will consider applications such as presenting a more realistic tactile sensation through the proposed tactile display by optimizing the voltage waveforms of both the electrovibration stimulus and the electrical stimulus.
